# Modeling repeated ordinal responses using a family of power transformations: application to neonatal hypothermia data

**DOI:** 10.1186/1471-2288-5-29

**Published:** 2005-09-14

**Authors:** Farid Zayeri, Anoshirvan Kazemnejad, Navid Khanafshar, Fatemeh Nayeri

**Affiliations:** 1 Department of Biostatistics, School of Medical Sciences, Tarbiat Modarres University, Tehran, Iran; 2 Department of Biostatistics, School of Medical Sciences, Tarbiat Modarres University, Tehran, Iran; 3 Department of Obstetrics and Gynecology, Tehran University of Medical Sciences, Tehran, Iran; 4 Department of Neonatology, Tehran University of Medical Sciences, Tehran, Iran

## Abstract

**Background:**

For analyzing a repeated ordinal response, it is common to use a multivariate cumulative logit model. This model may fit poorly, especially when a nonsymmetric response is available. In these cases, alternative strategies should be utilized.

**Methods:**

In this paper, we present a family of power transformations for the cumulative probabilities to model asymmetric departures from the random-intercept cumulative logit model. To illustrate this method, we analyze the data from an epidemiologic study to identify risk factors of hypothermia among newly born infants in some referral university hospitals in Tehran, Iran.

**Results:**

For hypothermia data, using this family of transformations and comparing the goodness-of-fit statistics showed that a model with the cumulative complementary log-log link gives us a better fit compared to a model with the cumulative logit link.

**Conclusion:**

In some areas, using the ordinary cumulative logit link function does not lead to the best fit. So, other link functions should be evaluated to discover the best transformation for the cumulative probabilities.

## Background

Hypothermia is an important cause of morbidity, and occasionally mortality, in the newborn [1]. In 1958, Silverman *et al. *[2] and in 1964, Buetow and Klein [3] reported the adverse effects of hypothermia on viability and hope for life in premature and low birth weight neonates. Low body temperature in newborns can lead to an increased rate of basal metabolism, peripheral vasoconstriction, decreased peripheral perfusion, tissue ischemia and finally metabolic acidosis [4]. Vascular changes in the lungs may result in decreased ventilation, increased demand for oxygen and worsening of respiratory distress [5]. Meanwhile, acidosis and hypoxia can predispose to pulmonary hemorrhage and disseminated intravascular coagulation (DIC) [4]. Hepatocyte ischemia affects liver functions and may cause indirect hyperbilirubinemia. In addition, the high metabolic rate leads to higher glucose consumption and hypoglycemia [5]. In many parts of the world, health personnel are not aware of the importance of keeping babies warm by simple methods such as drying and wrapping immediately after birth, avoiding harmful practices, encouraging early breast feeding and keeping newborns in close contact with their mothers [6].

Considering the prevalence of hypothermia experienced by Iranian neonates, and regarding that there is not adequate information about this health problem in our country, we decided to design an epidemiologic study to estimate the incidence rate and identify some of the most important risk factors of neonatal hypothermia in different referral hospitals of Tehran, Iran. In this longitudinal study, the body temperature of the newborns was measured repeatedly at several occasions. At each time of measurement, the ordinal outcome was defined as the severity of hypothermia for each newborn.

To analyze the dependence of a categorical response data on explanatory variables it is common to fit transforms of the probabilities by linear functions of parameters. In this context, the logistic transform is probably the one most commonly used. However, as for all models, it is tentative and therefore some consideration of adequacy is needed. If some non-logistic model gives a better or simpler fit, it is important to discover that. In this paper, we introduce a family of random-effects models to describe the relationship between repeated ordinal response data and a host of covariates. Using this family of statistical models, we are able to model asymmetric departures from the cumulative logit model. This approach is a simple and straightforward extension of the family of power transformations introduced by Aranda-Ordaz [7] to model asymmetric departures from the ordinary logistic regression model. We also develop the necessary computer program to obtain the maximum likelihood estimates of the model parameters.

## Methods

### The study of hypothermia in the newborns

The study of hypothermia was an epidemiologic research in some referral university teaching hospitals of Tehran, Iran. In this study, the researchers aimed to estimate the incidence rate of hypothermia and identify some of the most important risk factors of this health problem. To do this, a random sample of 900 newborns was selected in these hospitals from August 2003 to May 2004. After obtaining consent from the neonates' parents, the rectal temperature of the newborns was measured using a low-reading rectal thermometer at the following occasions;

i) Immediately after birth in the operating room

ii) After admission to the neonatal unit (levels I, II, III of nursery care)

iii) One hour after admission to the neonatal unit

iv) Two hours after admission to the neonatal unit

If a newborn was hypothermic, she/he was re-warmed according to WHO recommendations [6]. The ordinal response variable was defined as the severity of hypothermia at each occasion; 1 = moderate to severe hypothermia (temperature less than or equal to 35°C), 2 = mild hypothermia (temperature between 35°C and 36.5°C), 3 = normal body temperature (temperature between 36.5°C and 38°C). In addition, the following covariates were considered as the potential risk factors or risk indicators for neonatal hypothermia; sex (0 = male, 1 = female), weight (0 = more than or equal to 2500 gr, 1 = less than 2500 gr), gestational age (0 = more than or equal to 37 weeks, 1 = less than 37 weeks), environmental temperature at each time of measurement (0 = more than or equal to 27°C, 1 = less than 27°C, where 27°C was the mean temperature of the operating room and neonatal unit during the study), apgar score (a quick method of assessing the state of newborn infant. This score comprises five components: heart rate, respiratory effort, muscle tone, reflex irritability, and color, each of which is given score of 0, 1, or 2 [8]) immediately and five minutes after birth (0 = more than or equal to 8, 1 = less than 8) and cardiopulmonary resuscitation (CPR) (0 = not received, 1 = received).

Note that, the recorded outcomes for each newborn (severity of hypothermia at different occasions) are positively correlated ordinal observations, so convenient statistical approaches should be utilized to model the relationship between this response data and the described factors.

### Family of transformations for repeated ordinal response data

Suppose, in a longitudinal study, there are *T *occasions (times) of measurement, and the ordinal response at each time has *j *= 1,2,...,*J *levels. This ordinal response for *i*th individual (*i *= 1,2,...,*N*) at *t*th time of measurement (*t *= 1,2,...,*T*) can be denoted by *Y*_*it*_. Each individual has *T *covariate vectors *x*_*it*_, each of dimension *P × *1; the vector *x*_*it *_contains all the relevant covariates at time *t*, including time-dependent and time-stationary covariates. We also let *X_i _*= (*x*_*i*1_,...,*x*_*iT*_)' represent the *T × P *matrix of covariates for subject *i*.

In 1981, Aranda-Ordaz [7] introduced a family of asymmetric transformations for binary response data in the form of

*w*(*π*) = {(1-*π*)^-*λ *^-1}/*λ *    (1)

where 0 <*π *< 1 denotes the probability of success and *λ *is the transformation parameter. This seems to be a useful transformation when it is desirable to treat successes and failures asymmetrically. Using equation (1), one can denote the GLM form of this family as

log *w*(*π*) = *η *    (2)

where *η *= *β'X *is the linear systematic part of the model, and *β *is a vector of unknown regression parameters. Using equations (1) and (2), the family of asymmetric transformations for univariate binary response data can be written as

log{[(1-*π*)^-*λ *^-1]/*λ*} = *X'β *    (3)

Using simple calculations, one can show that for *λ *= 1 equation (3) reduces to the ordinary logistic model, while for *λ*→0 the complementary log-log model is obtained.

Now, we generalize the described family of power transformations to repeated ordinal response data. Suppose *π*_*itj *_= *pr*(*Y*_*it *_= *j *| *u*_*i*_) denotes the probability of ordinal response *j *for *i*th individual at time *t*. Now, using this definition and considering the equations (2) and (3), a family of random-intercept models for repeated ordinal response data can be defined as

log *w*(*γ*_*itj *_| *u*_*i*_) = *u*_*i *_+ *α*_*j *_+ *β'x*_*it *_    (4)

where *γ*_*itj *_= *pr*(*Y*_*it *_≤ *j *| *u*_*i*_) is the cumulative probability of response category *j *for individual *i *at time *t*. Here, *u*_*i *_denotes the random term for cluster *i*, *α*_*j*_'s are known as model cut-off points and *β *is the common *P *× 1 vector of fixed-effect regression parameters.

Using equation (4) for hypothermia data, the family of random-effects models can be written as

log{[(1-*γ*_*itj*_)^-*λ *^-1]/*λ *| *u*_*i*_} = *u*_*i *_+ *α*_*j *_+ *β*_1_*Sex*_*i *_+ *β*_2_*Weight*_*i *_+ *β*_3_*Gestational_age*_*i *_+ *β*_4_*Environmental_Temp*_*it *_+ *β*_5_*Apgar*1_*i *_+ *β*_6_*Apgar*5_*i *_+ *β*_7_*Multiple_preg*_*i *_+ *β*_8_*CPR*_*i *_    (5)

for *i *= 1,2,...,900, *t *= 1,2,3,4 and *j *= 1,2. Here, *γ*_*it1 *_= *π*_*it1 *_is the probability of being moderate to severe hypothermic for newborn *i *at time *t *(probability of being in the first category of ordinal response) and *γ*_*it2 *_= *π*_*it1 *_+ *π*_*it2 *_is the probability of being hypothermic (including mild, moderate or severe) for *i*th neonate at *t*th time of measurement (the probability of being in the first or second category of ordinal response). We also suppose that *u*_*i *_~ *N*(0,*σ*^2^), where *σ *is an unknown scale parameter which should be estimated in the model fitting process.

### Maximum likelihood estimators and computer programs

The model in equation (4) has *P *unknown fixed-effect regression parameters (*β*), *J*-1 unknown cut-off points (*α*_*j*_), and a parameter pertaining to the distribution of *u*_*i *_(*σ*, the standard deviation of the random-effect terms).

Consider the random-intercept model in equation (4). Assuming *η *= *u*_*i *_+ *α*_*j *_+ *β'x*_*it*_, it is easy to show that



and, therefore, the marginal probabilities can be computed by the following equation

*π*_*itj *_= *γ*_*itj *_- *γ*_*it*(*j*-1) _    (7)

To write the required likelihood function, one can form *J *indicator random variables *y*_*itj*_, where *y*_*itj *_= 1 if *Y*_*it*_*= j*, and *y*_*itj *_= 0 if otherwise. The marginal distribution of *Y*_*it *_is assumed to be multinomial (with sample size *y*_*it*+_*=*1), that is



Now, the necessary log-likelihood function for estimating the model parameters can be written as below



where *ψ *is a vector including all the unknown model parameters.

Solving the above score function in a random-effects model generally is not trouble-free, especially with an unknown transformation parameter. In this context, the procedure NLMIXED in statistical software SAS usually works well. By a user-friend programming, the likelihood function in equation (8) can be defined and then available estimating methods in this procedure help us to find the parameter estimates and related standard errors. For hypothermia data set, we used a *Dual Quasi-Newton *as the optimization technique and an *Adaptive Gaussian Quadrature *as the integration method. In addition, some useful goodness of fit statistics such as Akaike's Information Criterion (AIC) and Schwartz's Bayesian Information Criterion (BIC) are available in this procedure. A model with the smallest value of AIC or BIC shows a better fit compared to other random-effects models. To find more detailed descriptions about fitting the random-effects models, the interested reader can refer to Agresti [9]. In addition, the SAS code for fitting the random-intercept model in equation (5) is available in Appendix. The NLMIXED procedure estimates all the unknown parameters in the log-likelihood function. We denoted the unknown fixed-effect regression parameters by *b1, b2, ..., b8 *and the unknown transformation parameter (*λ*) by *b9*.

## Results

### Description of the data

The study sample consisted of 900 neonates (452 male and 448 female newborns). Of these, 298 newborns (33.1 percent) had low or very low birth weight (weight less than 2500 gr), and 323 newborns (35.9 percent) were preterm (gestational age less than 37 weeks). The mean temperature of the operating rooms and neonatal units was about 27°C (SD = 2.1). In addition, 726 neonates (80.7 percent) had apgar score more than or equal to 8 at the first minute after birth and 844 newborns (93.8 percent) had apgar score 8 or higher five minutes after birth. In this sample, the rate of multiple pregnancies was about 3 percent. Additionally, 63 newborns (7 percent) received CPR during the study. It should be noted that 42 hypothermic newborns (9 percent) died in a short period after birth, while this rate was about 2.7 percent (11 newborns) for the non-hypothermic neonates.

As mentioned before, for each newborn the ordinal response variable was the severity of hypothermia at each time of measurement. Table 1 shows the incidence rate and severity of hypothermia among these newborns, separately in four consecutive measurements. Summing over mild and moderate-severe rows in this table shows that 53.4, 13.7, 2.9 and 0.7 percent of these newborns were hypothermic, respectively at these four consecutive measurements.

**Table 1:** Severity of hypothermia among the sample neonates

		**Occasion**				
			
		**Time 1**	**Time 2**	**Time 3**	**Time 4**	**Total**
**Severity of Hypothermia**	**Normal**	419	777	874	894	2964
	**Mild**	329	105	20	4	458
	**Moderate-Severe**	152	18	6	2	178
	**Total**	900	900	900	900	3600

### Analysis of risk factors

To evaluate the fit of the illustrated model and to identify the significant risk factors of neonatal hypothermia, we first fit the described model in equation (5) with unknown transformation parameter in order to estimate this parameter and provide preliminary information about the appropriate link function for this data set. Table 2 shows the estimates, standard errors, p-values, and goodness-of-fit statistics. The estimate of transformation parameter, that is , shows serious departure from the cumulative logit model. In other words, since the estimate of transformation parameter is very close to zero, we can conclude that a model with the cumulative complementary log-log link (*λ*→0) seems to be more convenient for this data compared to a model with the cumulative logit link (*λ *= 1).

**Table 2:** Estimates from the longitudinal hypothermia data

	Unknown λ			λ = 1			λ→0		
Parameter	**Est**^†^	**SE**^‡^	**P**^§^	**Est**	**SE**	**P**	**Est**	**SE**	**P**

**α_1_/Cutoff 1**	2.932	0.256	_	4.212	0.308	_	3.611	0.235	_
**α_2_/Cutoff 2**	5.839	0.261	_	14.144	0.306	_	6.733	0.269	_
**β_1_/Sex**	0.118	0.126	0.346	0.585	0.562	0.298	0.230	0.206	0.264
**β_2_/Weight**	1.178	0.153	<0.001	3.005	0.773	<0.001	1.242	0.157	<0.001
**β_3_/Gest Age**^#^	0.351	0.174	0.044	1.224	0.654	0.061	0.249	0.112	0.025
β_4_/Env Temp*	2.976	0.501	<0.001	4.368	0.648	<0.001	2.247	0.511	<0.001
β_5_/Apgar1**	0.370	0.153	0.016	1.096	0.533	0.040	0.400	0.185	0.031
β_6_/Apgar5***	1.037	0.366	0.005	2.629	1.198	0.028	1.009	0.436	0.021
**β_7_/Multi Preg**^$^	0.802	0.352	0.023	1.659	0.805	0.039	0.776	0.336	0.021
**β_8_/CPR**	0.302	0.104	0.004	0.272	0.137	0.047	0.277	0.114	0.015
**σ**	3.584	0.395	<0.001	5.987	0.648	<0.001	3.977	0.410	<0.001
**λ**	0.031	0.013	0.020	_	_	_	_	_	_

**-2log.likelihood**		3882.6			3906.8			3889.5	
**AIC**		3904.6			3928.8			3911.5	
**BIC**		3972.7			3996.9			3979.6	

§ p for two-sided p-value

† Est for estimate of the model parameter

‡ Se for standard error of the estimate

# Gest Age for gestational age of the neonate

* Env Temp for environmental temperature

** Apgar1 for apgar score at the first minute after neonate's birth

*** Apgar5 for apgar score five minutes after neonate's birth

$ Multi Preg for multiple pregnancy

In the next step, we used the cumulative complementary log-log and logit link functions to model hypothermia data (Table 2). The obtained results tell us that there are substantial differences between the parameter estimates in these two models. Furthermore, comparing the goodness-of-fit statistics reveals that the model with the cumulative complementary log-log link gives a better fit compared to the model with the cumulative logit link function. This is not in contrast with the obtained results from the model with unknown transformation parameter.

Here, it should be noted that the fitted models did not lead to the same significant risk factors for hypothermia. Regarding to the column of p-values in Table 2 for the model with unknown *λ *and the model with the cumulative complementary log-log link, we can conclude that all the described factors, except sex, are significantly associated with hypothermia. But in the cumulative logit model (the model with *λ *= 1), gestational age of the neonates did not show significant effect on hypothermia.

## Discussion

Longitudinal studies are now widespread in many areas of medical research. The statistical analysis of these studies is usually difficult, especially when a repeated ordinal response is available [10,11]. In this context, generalized estimation equations methodology, introduced by Liang and Zeger [12], is a helpful strategy for analyzing repeated binary response data. In 1994, Lipsittz et al. extended this methodology to repeated categorical responses [13]. The choice of appropriate method for analyzing repeated categorical responses depends heavily on the aims of the study. Marginal and random-effects models are probably the most common approaches for the analysis of correlated categorical response data. Carrière and Bouyer presented helpful strategies for choosing marginal and random-effects models in longitudinal binary responses. They demonstrated that if the main goal of the study is to predict a mean prevalence of a specific disease over time by sex, age group or other characteristics, the marginal models are suitable. In contrast, if the goal is to study the individual risk factors for etiological considerations, the random-effects models are more appropriate because they allow adjustment on non-observed individual characteristics and a better understanding of the underlying mechanism [14].

In the present article, we introduced a family of power transformations to model the repeated ordinal response data. Depending on the main aim of the study, when a random-effects model is chosen for analyzing a repeated ordinal response, the first option is probably a model with the cumulative logit link function. Our main goal in the present study was to obtain more efficient estimates compared to those obtained using the cumulative logit link function. Nowadays, by using powerful statistical softwares such as SAS and S-PLUS, the fitting process is not too difficult. The required time for running process, even in large sample size data sets, is not more time-consuming compared to the ordinary random-effects models.

Supposing *J *= 2, the model in equation (4) reduces to a model for the analysis of repeated binary response data. In this situation, *Y*_*it *_is a scalar. Omitting the random terms *u*_*i*_, with *T *= 1 and *J *= 2, this approach reduces to the model presented by Aranda-Ordaz.

In our proposed model, assuming an unknown transformation parameter, *λ*, and estimating this parameter in the model fitting process is an appropriate strategy for checking asymmetric departures from the logistic model. If the estimate of the transformation parameter showed a serious departure from 1, then it can be concluded that the logistic transformation is not an appropriate option. For a given data, if the estimate of the transformation parameter is significant but not close to 0 or 1, then it can be concluded that neither the logit nor the complementary log-log link is appropriate. In this situation, one can fit the model in equation (4) with the estimated transformation parameter. Otherwise, if the estimate of this parameter is not significant or the standard error of the estimate is too large, the traditional methods of choosing the link function (for example, fitting the ordinary cumulative models with the common link functions such as logit, probit, complementary or negative log-log and then choosing the model with the best fit) may be preferable. To decide about the proper choice of transformation parameter for a given data set, an alternative strategy may be fitting this model with a sequence of values of *λ *and comparing the obtained goodness-of-fit statistics to determine a model with the best fit. In this context, drawing a graph of lambda versus common goodness-of-statistics (such as deviance) is a convenience approach for choosing a model with the proper transformation parameter.

As we mentioned before, the results of the present study showed that low birth weight and premature newborns, neonates with low apgar scores and those who received cardiopulmonary resuscitation had higher risk for being hypothermic. The same findings have been already reported in other research [15-18]. Moreover, the results of regression analysis revealed that the environmental temperature is significantly associated with neonatal hypothermia. It appears that newborns have higher risk for hypothermia when the operating room or neonatal unit temperature is not warm enough (in our study at least 27°C). This finding shows the importance of keeping the operating room and neonatal unit warm enough to reduce the risk of hypothermia among newly born infants. In general, theses results help us to train the medical care personnel for a better management of high risk newborn babies.

## Appendix

### SAS code for fitting the proposed random-effects model

data set1;

infile 'a:\hypothermia.dat';

y1 = 0; y2 = 0; y3 = 0;

if response = 1 then y1 = 1;

if response = 2 then y2 = 1;

if response = 3 then y3 = 1;

proc nlmixed qpoints = 100;

bounds i2>0;

bounds b9>0; *** b9 is the unknown transformation parameter,λ***

eta1 = i1+ sx*b1+ wt*b2+ ga*b3+ et*b4+ ap1*b5+ ap5*b6+ mp*b7+ cpr*b8+ u;

eta2 = i1+i2+ sx*b1+ wt*b2+ ga*b3+ et*b4+ ap1*b5+ ap5*b6+ mp*b7+ cpr*b8+ u;

p1 = 1-((1+ b9*exp(eta1))**(-1/b9));

p2 = (1+ b9*exp(eta1))**(-1/b9)) - ((1+ b9*exp(eta2))**(-1/b9));

p3 = (1+ b9*exp(eta2))**(-1/b9));

LL = y1*log(p1)+ y2*log(p2)+ y3*log(p3);

*** LL is the log-likelihood function ***

model response~general(LL);

estimate 'intercept2' i1+i2;

random u~normal(0, sigma*sigma) subject = case;

run;

## Competing interests

The author(s) declare that they have no competing interests.

## Authors' contributions

FZ performed the statistical analysis, interpreted the results and drafted the main body of the manuscript. AK was the chief coordinator of the study and participated in the drafting of the manuscript. NK participated in the data collection, the drafting of the manuscript and coordinated the communications. FN coordinated the medical part of the research, designed the questionnaire and cooperated in the drafting of the manuscript. All authors read and approved the final manuscript.

## Pre-publication history

The pre-publication history for this paper can be accessed here:


